# Navigating through trauma-informed care and behavioral treatment in young children with feeding problems

**DOI:** 10.3389/frcha.2026.1791744

**Published:** 2026-08-10

**Authors:** Meeta R. Patel, Ashley S. Andersen-Weber

**Affiliations:** 1 Clinic 4 Kidz, Sausalito, CA, United States; 2 Department of Pediatrics—Gastroenterology, Stanford University School of Medicine, Stanford, CA, United States

**Keywords:** ARFID, behavioral feeding treatment, feeding problem, failure to thrive, pediatric feeding disorders, trauma-informed care

## Abstract

Children may experience pediatric medical traumatic stress as a consequence of exposure to medical procedures, illness, or injury that are perceived as distressing, threatening, or otherwise aversive. In order to address the traumatic stress, trauma-informed care practices are warranted. The application of trauma-informed care practices has been widely used in healthcare for over 25 years; however, its integration into behavior-analytic practice has emerged more recently. Although trauma-informed care principles have informed practice across multiple areas of behavior analysis, relatively few studies have addressed their application to the treatment of pediatric feeding problems. Rajaraman et al. proposed four tenets of trauma-informed care that complement behavior-analytic practice as a whole: (1) acknowledge trauma and its potential impact, (2) ensure safety and trust, (3) promote choice and shared governance, and (4) emphasize skill building. In this paper, we describe a novel framework of the application of these tenets into behavioral feeding treatment. Specifically, we examine a balanced progression in which trauma-informed care and evidence-based interventions are integrated and implemented in tandem.

## Introduction

Trauma, according to the American Psychological Association (APA) is defined as an “emotional response to a terrible event like an accident, crime, natural disaster, physical or emotional abuse, neglect, experiencing or witnessing violence, death of a loved one, war, and more” (1). In addition, trauma can also be experienced after negative medical experiences. Pediatric medical traumatic stress (PMTS) according to the National Child Traumatic Stress Network refers to a child's psychological and physiological response to pain, injury, illness, or invasive medical procedures (2). In the PMTS framework, potentially traumatic events (PTEs) are used to describe exposures to medical experiences involving actual or perceived threat to bodily integrity, intense pain, or loss of control, including invasive procedures, life-threatening illness, and distressing hospitalizations (3, 4). These exposures are clinically significant not because they result in psychopathology, but because they may contribute to PMTS responses in a subset of children, depending on individual vulnerability, developmental stage, and contextual factors (4, 5).

It has been reported that 25%–30% of children who have negative health experiences develop posttraumatic stress symptoms (6). These symptoms may include avoidance (avoiding situations, people, or stimuli that remind them of the trauma; withdrawing from social situations), emotional responding (crying, irritability, temper tantrums) accompanied by a limited repertoire of effective responses to aversive private and environmental events, dependence on caregivers with limited autonomy, or overall deficits in social skills (7). However, it is important to note that each individual may experience PTEs differently and for some, psychological trauma may not occur.

For children with trauma histories, trauma-informed care (TIC) principles should be integrated across both assessment and treatment to promote safety and avoid retraumatization, with an overarching goal of improving well-being of the individual (5). Trauma-informed care refers to healthcare services that focus on past experiences of trauma when developing a treatment plan, rather than just focusing on the immediate presenting problem (8). A trauma-informed care approach recognizes that a child's prior PTEs are central to understanding their current social, emotional, and physical well-being. It also emphasizes that trauma may shape emotional responses, behavior, and physiological functioning, and that this context should be integrated into clinical formulation and treatment planning to ensure that care is responsive to each individual's trauma history (5, 9). Key features of trauma-informed principles include promoting physical and psychological safety, fostering trust and transparency, increasing opportunities for child choice and assent, supporting collaboration with caregivers and stakeholders, and emphasizing strengths-based skill development (10, 11).

Children who display feeding problems, such as tube dependency (due to total food refusal), poor oral intake, food selectivity by type or texture, or liquid dependence, may experience PMTS as a result of their unique medical or experiential histories. For example, children who have had extended neonatal intensive care (NICU) admissions at birth or extended hospital admissions are more likely to have feeding problems because of invasive medical procedures near or around the mouth, such as several attempts of intubations or nasogastric (NG-) tube placements under physical restraint, to name a few (12, 13). As a result of these negative experiences, children may learn to avoid eating altogether or eat very little by mouth. It is also likely that certain gastrointestinal issues, such as constipation, gastroesophageal reflux, or food allergies, which can cause recurrent vomiting or pain during or after eating, may result in avoidance of eating all or certain foods (14). In addition, a single episode or recurring episodes of choking and gagging could also alter a child's willingness to eat certain foods or they may avoid all solid foods and become dependent solely on liquids (15). Furthermore, children with certain diagnoses, such as autism spectrum disorder, may be sensitive to various sensory stimuli (e.g., flavor, texture, smell) which may lead to certain foods being avoided; that avoidance may be generalized to other foods, further restricting their diet (16). Lastly, children may develop feeding challenges as a result of high-pressure feeding situations that occur due to parental stress, and consequently may learn to avoid eating or develop negative responses toward food (17–19).

A child's response to these negative experiences may result in a variety of refusal behaviors when presented with food or liquid, such as pushing the food or liquid away, turning their head when food or liquid is presented, and emotional responding during mealtimes. Even when the child is no longer in pain or discomfort, these behaviors may persist because of the child's reinforcement history. That is, how individuals in the child's environment (often parents or caregivers) respond to these behaviors impacts whether these behaviors will continue to occur. The literature has shown that these behaviors are often maintained by negative reinforcement in the form of escape (20). Following these behaviors, the caregiver may take away the food, so the likelihood of these behaviors occurring in the future increases. Therefore, a child may learn to engage in similar behaviors in the future to avoid eating.

Although there has been limited research on PMTS in children with feeding problems, it is important to understand the role trauma plays in the treatment of these problems. Guvenek-Cokol et al. (7) presented a case study of PMTS in a 5-year-old girl who was admitted for abdominal pain and total by mouth (PO) refusal. The child was placed through extensive testing, NG-tube placement, and total parenteral nutrition (TPN) prior to being admitted to another hospital. During her first admission, the child was present while providers and caregivers discussed the course of treatment, including potentially frightening procedures, without being directly involved in these discussions. The parents reported that the child was more withdrawn and more oppositional after the first hospital admission. These authors emphasized the need for a multidisciplinary treatment approach that involved gastroenterology, nutrition, behavioral psychology, psychiatry, child life specialist, social work, and occupational and speech therapy, which this child was not getting prior to her admission to the second hospital. This child was previously only being treated by medical professionals and the psychological piece was not being addressed until she was admitted to the new hospital. In this study, they evaluated a consistent, supportive, and familiar environment for the child and developed a behavior plan which included time-limited food offerings, choice, no food offerings outside of scheduled times, limiting fun activities to meals, and verbal praise for acceptance. This treatment approach aligned with trauma-informed care practices, and as a result of this care plan, the child was able to eliminate NG-tube feedings and transition to 100% PO feeds by discharge. Furthermore, it is likely that if trauma-informed care practices were utilized from the onset of treatment in the first hospital, additional psychological trauma may have been avoided.

Trauma-informed care is well adopted and utilized in patient care in most healthcare services (21); however, its adoption in behavior-analytic practice has been relatively recent. Rajaraman et al. were the first to discuss how a trauma-informed care framework can be utilized within practice to avoid harm and re-traumatization, urging practitioners to consider an individual's trauma history when developing behavioral assessments and treatments (11). Further, trauma-informed care practices within behavior analysis could promote the individual's dignity and treatment acceptability. It is important to note that trauma-informed care does not refer to a specific set of procedures but rather a set of values for how services should be delivered (10). Austin et al. discuss how some procedures may be less aligned with trauma-informed care, but can potentially be delivered in a compassionate manner (10). They further state that dismissing procedures as trauma-inducing without understanding the side effects could potentially disregard effective treatments.

Although interest in trauma-informed care within behavior analysis has grown, the empirical evidence base remains limited. Most published work consists of conceptual discussions, practice recommendations, and preliminary investigations rather than controlled outcome studies (10, 11, 22). Current research highlights the need for operational definitions of trauma-informed behavior-analytic practice, validated measures of implementation fidelity, and rigorous evaluations of treatment outcomes (22).

Trauma-informed care is especially important when working with children with feeding problems because oftentimes refusal behaviors emerge due to negative experiences with aversive procedures around the mouth, pain or discomfort, or both. Understanding a child's unique history may help develop a treatment plan that takes into account traumatic experiences which may result in a treatment that is more preferred or implemented with higher integrity. Furthermore, it may establish eating-related behaviors as more reinforcing, which may contribute to the long-term maintenance of treatment gains. In addition, understanding the child's extensive history may be critical in developing a starting point for feeding treatment that takes into account PTEs.

Although trauma-informed care practices seem most relevant when working with children with feeding problems who may have experienced a PTE in the past, many clinicians feel ill equipped to incorporate trauma-informed care practices in treatment because of a lack of training and trauma-informed care not being clearly defined or practiced in behavior analysis (23). Further, Wheeler et al. (24) found that although the majority of clinicians reported being moderately to very comfortable working with individuals who may have had adverse experiences, they also reported little to no coursework, hands-on experience, or consultation on trauma-informed care. More recent research has demonstrated that the use of checklists and goal-setting procedures increased the implementation of trauma-informed practices by behavior analysts in school settings (25); however, application of these procedures remains limited.

It is important to outline how trauma-informed care strategies could be incorporated in practice, especially with children with feeding problems. Examining these past events could potentially help build more effective treatments. However, it is also important to note that feeding problems can pose a serious health risk; therefore, one must know how to navigate through utilizing trauma-informed care and providing the most efficacious treatment for the presenting problem. The purpose of this paper is to describe a novel framework for applying the tenets outlined by Rajaraman et al. (11) to behavioral feeding treatment, with the goal of integrating trauma-informed care practices with empirically supported feeding interventions.

## Review of behavior-analytic feeding treatment

Behavior-analytic treatment for feeding focuses on establishing the efficacy of treatments using single-case design. The initial goal of behavior-analytic feeding treatment is to develop a plan to increase consumption, decrease refusal behaviors, and teach appropriate feeding skills (26). The long-term goal is for the child to develop healthy food preferences and eat nutritionally balanced meals in a variety of locations without a structured treatment protocol. To achieve these therapeutic outcomes, assessment of the feeding problem is critical.

A functional analysis identifies environmental variables responsible for the maintenance of food refusal (27). These refusal behaviors at the onset can be a result of PTEs in that, in the past eating, drinking, or both were paired with negativity (e.g., pain, discomfort). Further, for an event to be considered traumatic, the individual would also need to display a pattern of observable behavioral, emotional, and physiological responses, such as re-experiencing, triggered by related stimuli, avoidance of associated situations or people, and heightened physiological responding that impact day-to-day functioning (11). Over time, these refusal behaviors may be reinforced and continue in the child's repertoire due to a learning history that is established and maintained by the environment. Understanding the stimuli or stimulus conditions that evoke these behaviors can help us understand a starting point for treatment. As clinicians, if we can understand why and what the child is avoiding in the eating situation, we can alter those variables to establish a more effective treatment. Antecedent assessments that assess different stimuli that may evoke refusal behaviors can be useful to clinicians when developing treatments that are trauma-informed; however, this research is still in its infancy (28, 29). Another assessment tool often used in treating feeding problems is a food preference assessment that provides a hierarchy of food preferences and allows the clinician to determine food selection based on the results of the assessment (30–32).

A treatment that has been shown to be efficacious in treating feeding problems is escape extinction, in which a child is no longer allowed to avoid eating, either by nonremoval of the spoon where the spoon remains close to the mouth until the bite is accepted or some form of physical guidance (33). Escape extinction procedures are effective likely because these treatments address the escape function of the refusal behaviors. These procedures may be paired with temporary increases in behavior, but often these behaviors subside quickly and acceptance increases (34). Although escape extinction has the most empirical support, there has been more hesitation from practitioners to use this treatment due to ethical concerns with regard to choice and assent (35). Furthermore, it is a treatment that may be challenging to implement with older children and must not be implemented by those who have limited training working with children with feeding problems.

Because feeding refusal behaviors are often maintained by negative reinforcement (20), it is likely that some form of escape extinction may be necessary for some patients for whom the feeding problem is severe, but it is important to understand how escape extinction, if needed, can be implemented while still considering the child's unique trauma history. We propose in this paper that these two are not mutually exclusive, and that for health considerations, escape extinction may be necessary if done within a trauma-informed care framework if other treatments have been exhausted or more immediate behavior change is needed to maintain a child's health. It is important to understand whether escape extinction qualifies as a PTE, or may be influenced by prior PTEs, and how trauma-informed care principles may affect the implementation of this procedure.

There are no studies to date or long-term outcomes that show that escape extinction can be avoided in children with complex medical histories who display severe feeding problems or that assent can be honored while still achieving meaningful outcomes in those cases. Although escape extinction and other behavioral feeding treatments have strong empirical support for increasing consumption and reducing food refusal, there is limited systematic evaluation of long-term psychosocial outcomes, including emotional distress and family quality of life, following behavioral feeding treatment (36, 37).

In order to incorporate a child's trauma history, positive reinforcement may be critical because it is likely that positive reinforcement can decrease or circumvent negative side effects (e.g., increases in refusal behaviors, increases in emotional responding) when escape extinction is necessary (38, 39). Unfortunately, reinforcement-based procedures alone, either differential reinforcement (access to a tangible item/activity after a bite is consumed) or noncontingent reinforcement (access to tangible item/activity throughout the meal), have been less effective in treating feeding problems (38, 39); therefore, more emphasis on antecedent-based procedures is warranted, along with evaluating patients' trauma history.

Antecedent-based feeding treatments involve changing the environment prior to food being presented to decrease the value of escape as a reinforcer, and therefore decrease the likelihood of refusal behaviors occurring. For example, in the feeding context, antecedents that can be manipulated may involve food type, texture, presentation format, bite size, and bite number. Furthermore, other antecedents that can be manipulated in the feeding context may involve different seating arrangements, feeding locations, and feeding utensils, to name a few. More specifically, the antecedent-based treatments that have empirical support include fading (40), shaping (41), modeling (42), high-probability instructional sequence (43), chaining (44), and visual supports (44). However, the long-term efficacy of antecedent-based treatments alone is less clear, but there is more empirical support on their effectiveness when combined with positive reinforcement, escape extinction, or both (33).

Although trauma-informed care practices may be incorporated into behavioral feeding programs, particularly within intensive multidisciplinary settings, this information is not consistently documented in the published literature, nor has a systematic framework been established. Proctor et al. (45) provided detailed guidance on the assessment process and the role of multidisciplinary collaboration in feeding intervention; however, trauma was not explicitly assessed or evaluated as part of the clinical case study. More broadly, research examining the role of trauma history in the clinical management of pediatric feeding problems remains limited. A notable exception is Demchuk et al. (46), who integrated a patient's trauma history into treatment planning by evaluating a continuous reinforcement procedure in conjunction with escape extinction, while deliberately avoiding physical prompting given the child's history of physical trauma.

Moreover, there have been two published studies examining trauma-informed care practices in the treatment of food selectivity without the use of escape extinction for four children with autism who did not have a complicated medical or trauma history (47, 48). These authors used choice with regard to food item and what to do with the food (e.g., touch, smell, taste) for each trial and positive reinforcement was delivered on a gradient, based on engagement with the food. Assent was also assessed throughout the treatment which involved allowing the child to leave and come back to the treatment table at any time. The participants in these studies were successful at trying 3–6 novel foods with the shaping procedures, but it was unclear if they were in fact eating age-appropriate, nutritious portions at the conclusion of the study because a nutritional analysis was not provided. Furthermore, long-term follow-up data were not provided. Thus, the social significance of behavior change was unclear. It was also unclear if these procedures could generalize to participants with an extensive medical history who display total food refusal, where avoiding eating altogether may be more reinforcing than obtaining any other reinforcement for participation. The participants in these studies were children with autism who had functional communication, but it is unclear if these procedures would generalize to children with more significant cognitive delays who may have deficits with regard to language and choice-making abilities or younger children who have not yet developed choice-making skills.

Furthermore, across these studies, researchers identified positive reinforcers that were able to compete with escape as a negative reinforcer, particularly when escape was provided on a noncontingent basis. In other words, children were more likely to assent to treatment when doing so produced access to preferred stimuli or activities, even though they had the option to participate or refuse. However, treatment progress may be limited for some children when positive reinforcers (e.g., attention or tangible items) are not available to outweigh the reinforcing value of escape, especially when escape remains available for refusal behaviors. In such cases, withdrawal of assent may be more likely because escaping the treatment context functions as a more potent reinforcer than accessing preferred items or activities within that context.

Gover et al. (48) incorporated these trauma-informed care strategies in the treatment of food selectivity in one autistic child. However, there is limited data on how these tenets can be utilized to ensure long-term success with children with a variety of feeding challenges. It is important to note that previously published studies may have incorporated all or some aspects of these tenets; however, because that information is not always shared in publication it makes it difficult for clinicians to know how to navigate through trauma-informed practices for behavioral feeding treatment. Further, due to escape extinction being a treatment that has the most robust outcomes, it is important to understand how and when it can be used safely. Below we propose recommendations on how these strategies can be employed for children both with total food refusal and food selectivity being treated in a variety of settings. Furthermore, we present areas of future research where feeding treatment can be evaluated using trauma-informed care practices while ensuring long-term treatment gains using evidence-based treatments that use both antecedent- and consequence-based procedures. There is empirical evidence in the use of behavioral treatments for feeding problems; however, the efficacy of these treatments within a trauma-informed care framework is unknown since this information is not typically reported in published studies.

## Proposed framework of trauma-informed care in behavioral feeding treatment in young children

**Figure 1:**
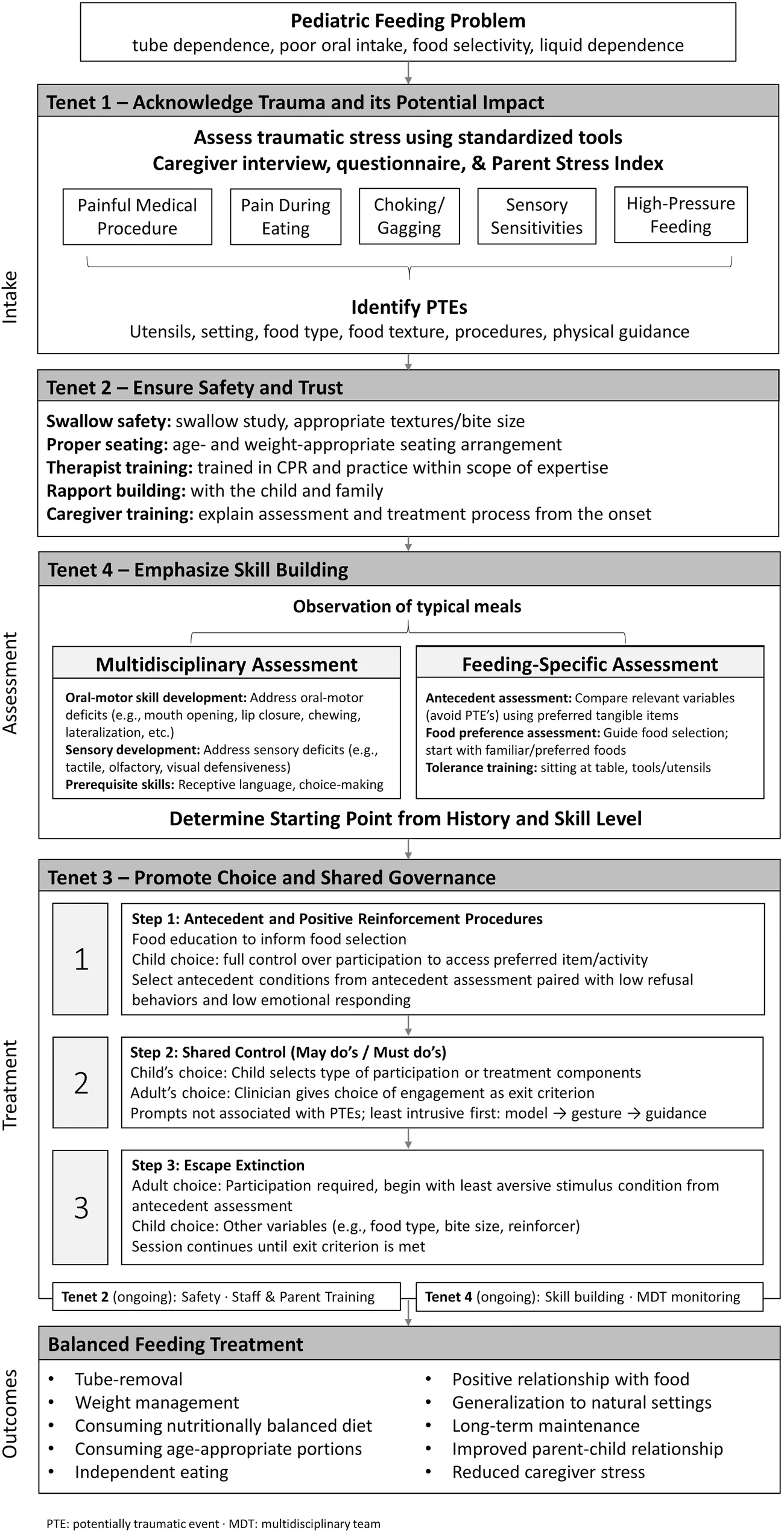
Trauma-informed care framework for behavioral feeding treatment.

Rajaraman et al. (11) proposed four tenets of trauma-informed care that complement behavior-analytic practice in general: (1) acknowledge trauma and its potential impact, (2) ensure safety and trust, (3) promote choice and shared governance, and (4) emphasize skill building. These tenets are discussed below within the context of behavioral feeding treatment. Figure 1 summarizes this novel framework that integrates trauma-informed care principles across the continuum of care, from intake and assessment through treatment implementation and treatment outcomes for pediatric feeding problems in children under the age of 12.

### Acknowledge trauma and its potential impact (intake)

To acknowledge trauma, clinicians should inquire into past events when developing treatment and recognize that individuals may respond differently because of those past experiences (11). Standardized and validated tools to assess traumatic stress may be a useful starting point to identify PTEs (49). However, this should only be conducted by trained professionals and not necessarily by a behavior analyst with limited training (50). Further, open-ended interviews to understand the child's unique history and to determine any potential negative past experiences (48) can also be essential. These interviews are often part of standard practice in intensive multidisciplinary feeding programs (51); however, validated traumatic stress measures may be less common. Standardized tools to assess trauma and a complete medical and feeding history would allow a clinician to determine potential past aversive stimuli that could affect present behavior.

It is also important to understand any medical procedures associated with negativity (e.g., pain or discomfort). For example, a child who has had aversive stimuli around or near the mouth, such as nasogastric tube placement or recurrent intubations, may respond differently to any stimuli coming to their mouth, including utensils (like a spoon). Feeding tools may function as aversive stimuli that resemble PTEs. Therefore, acknowledging this history may lead clinicians to develop an alternative starting point for treatment.

The same may be true for children who display food selectivity, in which understanding the child's consumption history can guide treatment. There is a large body of research in the food science literature that has evaluated why individuals eat what they eat based on sensory stimuli of foods (52, 53). Children who engage in food selectivity may reject certain foods due to heightened sensory awareness (16, 54). Therefore, even before food is presented, how children respond to sensory stimuli outside of mealtimes may need to be addressed by an occupational therapist (OT) for some children.

A child's unique history should be evaluated when developing a treatment plan. For example, if a certain food or food groups were mentioned as causing choking, gagging, or vomiting, those should be avoided at the onset of treatment because they may be paired with PTEs. A clinician in scrubs and gloves may also resemble PTEs. If a child engages in high levels of emotional responding every time they see a person in scrubs with gloves, it may be important that the treating clinician eliminate the use of gloves and have an extensive hand washing protocol as an alternative and potentially wear the child's favorite color of regular clothing instead.

Furthermore, when obtaining the child's history, the clinician needs to find out whether any physical management strategies in the past caused distress similar to Demchuk et al. (46). It is likely that if a child does not have a negative history associated with physical prompting, it is something a clinician may be able to use after attempting less intrusive procedures first, if needed. It would not be appropriate to deem all physical guidance procedures potentially retraumatizing if such a history was not identified as a PTE during the interview. It is important that the right questions be posed in an interview or a questionnaire. For example, if physical guidance was used, how was it used, when was it used, and what was the child's response? This information will allow clinicians to determine if physical guidance would be appropriate for a given child.

In addition to acknowledging the child's trauma history, it is also critical to understand caregiver anxiety and stress when developing a treatment plan. It may be helpful for the family to fill out a parent stress questionnaire prior to treatment (55). For example, if the caregiver is anxious about weight gain, then initial treatment goals may focus on volume initially rather than variety or self-feeding to alleviate those concerns. Involving caregivers from the start of feeding treatment may ease further stress and anxiety by empowering them with the skills to successfully feed their child. Furthermore, if the caregiver has other mental health concerns, these may need to be addressed outside of feeding therapy using a multidisciplinary approach. In addition, it may be critical to understand the entire family system, including siblings or grandparents, before treatment is developed because they may be delivering consequences that may be maintaining refusal behaviors and may need to be involved in training.

### Ensure safety and trust (intake)

Ensuring safety refers to creating an environment that is safe both emotionally and physically for the child. First, a modified barium swallow study should be performed to assess swallow safety and confirm that, when a swallow is elicited, the food or liquid is directed into the esophagus and stomach rather than the airway, thereby reducing the risk of aspiration (56). Furthermore, the swallow study may help determine the most appropriate bite size, food texture, and liquid consistency to optimize swallowing safety.

Another aspect of safety when it comes to feeding is proper seating arrangement. In the Gover et al. (47, 48) studies, the children were allowed to leave the table when they chose; however, from a safety perspective it may not be appropriate to leave the table if food is still in the mouth due to choking hazards. Similar to how children are required to sit in a car seat buckled for safety, regardless of if they want to sit or not, eating while seated may also need to be required. The seating arrangement in feeding treatment is critical for the safety of the child. A 90-90-90 position where a child's hips, knees, and ankles are all at 90 degree angles with foot support is recommended by speech and language pathologists (SLP) (57). Seating should be based on the child's height, weight, and medical status. For example, for children who have a history of aspiration, low muscle tone, or a similar diagnosis, a high back chair with a lap belt or a 5-point harness to avoid aspiration would be warranted. This may be true for children with oral-motor deficits as well to ensure that they are safely positioned while learning the skills to correctly manage food in the mouth. Safe seating has been stressed in previous research from clinic- and hospital-based feeding programs; however, it is important that this precaution be used in all environments that feeding treatment is being implemented.

Finally, to ensure safety, clinicians working with feeding problems must be trained in CPR and first aid and should not practice outside of the scope of their expertise. This is likely a requirement for all staff, in clinic- and hospital-based feeding programs; however, CPR and first aid training is important in all environments that children with feeding problems are served. Feeding intervention requires treatment with a multidisciplinary team so these safety precautions can be assessed throughout treatment.

The need for multidisciplinary involvement holds even for children who display food selectivity; it could be possible that there is an oral-motor skill deficit, sensory deficit, or emergent medical challenges, but these may not be identified by the behavior analyst alone, as these areas are typically not within a behavior analyst's area of expertise unless they've received specialized training. These deficits need to be addressed before treatment is initiated and evaluated throughout treatment by a multidisciplinary team to ensure safety. Board certified behavior analysts must review the ethical code of the Behavior Analyst Certification Board (BACB) (58) before proceeding with feeding treatment because it is a specialized population which requires the expertise of different disciplines. This may also be a useful document for clinicians working with children with feeding problems outside of the United States and are not certified.

In addition to safety, rapport building is also a critical aspect of the assessment phase (59, 60). Trust is built when a reliable relationship between the child and a clinician is established by way of the child readily interacting with the clinician independently (11, 61). Gover et al. (48) refers to ensuring safety and trust in feeding treatment as allowing assent to be withdrawn at any time and honoring choices. Although choices and assent are important in feeding treatment, the clinician must actively try to obtain that trust beyond allowing the child to make treatment decisions. Further, one may argue that trust can be built when a clinician sets boundaries and is consistent. Krauss et al. (62) discuss the importance of building trust with young patients and present a framework on how trust can be established in a medical setting. Similarly, in feeding treatment, ensuring safety and trust should first start with building a rapport with the child outside of the feeding situation.

Patel et al. (63, 64) are the only studies to date to discuss the importance of rapport building prior to and during feeding treatment. Rapport building would be initiated by the clinician prior to treatment development. After the caregiver interview or questionnaire, the clinician would be more equipped to understand the child's likes and dislikes. From this information, the clinician can set up play-only sessions in the child's safe environment (e.g., home, park) with preferred toys and activities to pair that clinician with positive reinforcement from the start. If treatment is conducted in a clinic or hospital, this relationship building may need to occur in a playroom or a location outside of the treatment room. Once that relationship is built, the child may be more willing to participate in feeding therapy.

The initial goal is to meet the child at their current level of responding for trust to continue to build. If the child has built trust with the clinician, then it is likely that the child may be more willing to cooperate with both feeding and non-feeding requests. This may not be possible if the child has the ability to withdraw assent at any point. Although choice and assent should be incorporated into treatment, it may be more powerful for the clinician to teach the child what it means to make an appropriate choice, rather than to allow assent to be withdrawn.

Moreover, building trust with caregivers or stakeholders in the assessment phase is critical (61). Therefore, explaining the assessment and treatment process and providing necessary training will allow for that trust to be built, along with incorporating caregiver input as was done in the Gover et al. (47) study. Because caregivers will be the change agents in the natural environment, including them throughout the assessment and treatment process using a family-centered approach may allow for that trust to solidify (65).

### Emphasize skill building (assessment)

In the Gover et al. (48) study, skill building was referred to as shaping different pre-consumption behaviors (e.g., looking at the food, touching the plate, touching food, touch to lips) to eventually lead to consumption. However, these behaviors do not necessarily capture a skill deficit per se, but are behaviors related to cooperation with eating instruction. It is likely that the child knows how to engage in these behaviors but is choosing to participate or not.

Observations of typical meals in the home environment are essential to assess actual skill deficits during the assessment phase, both at the multidisciplinary and feeding-specific levels. At the multidisciplinary level, assessments by an SLP can focus on oral-motor skill development related to eating and assess prerequisite skills, such as the child's receptive language level and choice-making repertoire.

The results of the swallow study may guide the SLP on how swallowing can be strengthened with exercises or using different compensatory strategies (e.g., specific vessels, thickened consistencies, positioning). Furthermore, opening the mouth wide to accept food or liquid and lip closure on utensils (66) should also be assessed prior to feeding treatment because without these skills it would be challenging for children to consume food and eventually do so efficiently. In addition, the child's oral-motor status should be evaluated by an SLP to determine the appropriate texture and consistency to initiate treatment based on skill level. If deficits are identified regarding chewing, it would be important for an oral-motor program to be developed to teach these skills before targeting consumption, and to teach these skills, physical prompting may be warranted (67).

Building the child's repertoire of eating behaviors may decrease the effort required to consume food, allowing them to contact the naturally reinforcing properties of eating. However, if the child chooses not to participate in skill development, then the child would not contact the automatically reinforcing properties of eating or contact positive reinforcement for eating, and the child would continue to experience eating as being negative and effortful. Therefore, even when it comes to skill building, exercises must be broken down into small steps so the child can contact reinforcement. This is an opportunity where choice can be incorporated where the child decides the order of the exercises, but the choice should not be to participate or not because skills must be built to progress towards long-term meaningful feeding outcomes.

At the multidisciplinary level, an OT may also evaluate sensory sensitivities that affect consumption. For example, assessing tactile, olfactory, and visual defensiveness may assist in treatment development. If a caregiver reports the child refuses to walk on grass, feel tags on clothes, or touch wet things, that should be addressed by an OT prior to initiating intensive feeding treatment because this sensitivity may generalize to food engagement. For example, if children show sensitivity to certain sensory stimuli outside of the mealtime context it may affect the child's willingness to accept or even touch food (68).

Before treatment can be initiated, feeding-specific assessments should be conducted where all potentially relevant antecedent variables (based on the child's individual history) are evaluated to align with a trauma-informed care approach. During antecedent assessments, data should be collected on acceptance, refusal behavior, emotional responding, and gagging with different antecedent conditions (e.g., utensils, food type, texture) while avoiding PTEs. The starting point for treatment should be identified by conducting assessments possibly within the context of positive reinforcement where access to preferred items is noncontingent, meaning freely available throughout the session (28, 29). Including preferred toys or activities during assessments would likely pair positive reinforcement with the feeding context in an attempt to keep refusal behaviors and emotional responding low so we can evaluate other variables that may be affecting eating. Likely, when refusal behaviors are high in a feeding context, the child may not respond at all during an assessment; therefore, the feeding problem would not be assessed effectively (61), so positive reinforcement during assessment may be useful.

Further, food preference assessments can guide food selection for children with food selectivity. To incorporate trauma-informed care practices for children with food selectivity, it is critical to understand the child's current preferences to help select foods in treatment. For example, assessing the sensory properties of the preferred foods prior to treatment may help identify other foods that share similar properties for initial food targets. In addition, for children with food selectivity who demonstrate advanced language and decision-making skills, it may be appropriate to involve them in selecting target foods for treatment. For children who are unable to make choices independently, intervention may first focus on developing choice-making skills prior to initiating feeding treatment. Alternatively, choices may be structured such that the adult preselects options that align with the child's nutritional needs. For example, the child may be permitted to choose a specific type of fruit, but not to opt out of including fruit in the meal.

Because many of these patients have experienced negativity with eating (e.g., gagging, choking, vomiting, allergic reaction), even before food can be introduced, the therapist may want to implement tolerance training after sufficient rapport has been built. First, the child may simply sit at the feeding table and play with preferred toys if the child is not already doing this, and over time the clinician adds more feeding-specific instructions, such as working on acceptance of the hand around and near the face or mouth (69), acceptance of tools in the mouth (e.g., finger, Nuk brush, chew tube) and eventually a spoon or cup (70).

### Promote choice and shared governance (treatment)

Promoting choice and shared governance refers to incorporating choice and autonomy in treatment. In both Gover et al. (47, 48) studies, children assented to treatment and were allowed to leave the treatment sessions at any point. Feeding problems are most often treated in younger children; therefore, formal assent procedures may not always be required. Furthermore, some children may not be able to assent to treatment due to language deficits or limited decision-making capacity. Similar to medical procedures that are mandatory for the child's health, assent may not be appropriate in those situations because it is not a choice to have a medical procedure or not due to health risks. Although research guidelines, depending on geographical location, indicate that assent should be obtained from children approximately seven years of age and older (71) and legal consent for treatment is typically reserved for adolescents, incorporating assent procedures may still be clinically valuable when treating less severe feeding problems (47).

Also, Gover et al. (48) allowed the child to select the foods to target in treatment as well as selecting what he wanted to do with the food. Choice has also been incorporated in the existing feeding literature (72–74); however, more emphasis may be needed on food selection for treatment to improve the efficacy of choice. There are currently no published studies that evaluate systematic food selection in treatment; however, in clinical practice, we often start with foods that are familiar first, similar to a food chaining model (75) and to avoid foods that may have been paired with negativity in the past. It may be helpful to start treatment with foods the child used to eat as long as those foods were not paired with PTEs. It is likely that more similar foods can produce lower levels of refusal behaviors and emotional responding, and possibly less gagging than foods that bear no resemblance to preferred foods.

Offering choices of food items can support autonomy; however, without proper knowledge of the foods, it may be difficult for the child to make an informed decision on which foods to target in treatment. Therefore, teaching the child about the different sensory stimuli of food and nutrition education can be a useful tool in increasing consumption (76, 77). For example, teaching the child to identify and label colors, flavors, and textures using familiar foods builds a verbal repertoire that provides context for novel foods and may reduce uncertainty and unfamiliarity when new foods are presented. Food education has shown promising outcomes in research outside of behavior analysis (76, 77); however, it may not be appropriate for children with limited language. Food selection in previous behavioral feeding research has typically been arbitrarily selected or selected based on caregiver preference or by a dietitian, which may still be appropriate for children who cannot make choices. However, familiarity or similarity of foods should still be evaluated when selecting food targets at the beginning of treatment.

Choices with regard to food selection, reinforcer selection, or even the type of feeding treatment to implement are great options to promote autonomy. However, it is also important that the efficacy of treatment is maintained while incorporating choices. If choices are not producing meaningful outcomes with regard to age-appropriate consumption of a variety of foods in all food groups, the choice arrangement may need to be altered. For example, once a choice has been made, that choice is no longer available, and reinforcement contingencies may be altered (47, 48). Terminating the session solely based on the child's choice may not always be effective long-term, especially if avoidance of eating is more reinforcing than any other secondary reinforcer.

The decision to initiate treatment should be individualized for each child, taking into account their unique history and current skill repertoire. However, when increases in participation are not observed following antecedent-based interventions, the implementation of positive reinforcement, or both, the use of shared control procedures may be warranted. Shared control, in which the child is provided with both “may do” (child-selected) and “must do” (adult-selected) options, may be appropriate in the context of feeding interventions (30, 73). Given the potential for severe medical consequences associated with pediatric feeding problems (e.g., dehydration, poor weight gain, constipation, or reliance on enteral feeding), it may not be appropriate to provide complete autonomy regarding food consumption. Nonetheless, opportunities for choice should be incorporated whenever feasible and clinically appropriate. For example, allowing a child to refuse all intake, leave the mealtime setting, or restrict intake to a single food group may pose significant health risks and therefore may not be in the child's best medical interest.

Accordingly, antecedent variables should be systematically implemented in conjunction with reinforcement contingencies prior to the implementation of more intensive procedures. If these components alone do not yield clinically meaningful improvements, the establishment of an explicit exit criterion may be appropriate. Such criteria may require a predefined level of interaction with the presented food (e.g., touch, lick, or bite) or another target response prior to termination of the session (79). Although the child may be permitted to select the manner in which participation occurs, nonengagement would not be an available option. Therefore, assent may be overridden at this point where meaningful but bounded choices would be available, rather than the option to refuse participation. If independent engagement does not occur reliably, graduated prompting procedures may be necessary, provided they do not resemble PTEs. In such cases, intervention should begin with the least intrusive prompts, such as modeling, gestural prompts, and then physical guidance as clinically indicated.

Most often in the behavioral feeding literature there is a meal cap where the meal is terminated when the time is reached; however, that too could potentially reinforce avoidance behaviors if meals terminate without some form of food engagement. Furthermore, allowing the child to leave the session as they choose (47, 48) could also further reinforce avoidance behaviors. That being said, choice and shared governance are critical when trying to promote trauma-informed care practice. Therefore, providing a balance between allowing choice and having the child contact positive reinforcement may require some form of escape extinction to achieve long-term treatment outcomes.

If shared control does not produce sufficient progress, then some form of escape extinction may need to be implemented, in which participation is required but always begins with the least aversive stimulus condition identified in the antecedent assessment, similar to the extinction procedure as described by Tarbox et al. (80). It is possible that initiating treatment with the least aversive stimulus condition (e.g., empty spoon) would minimize or eliminate refusal behaviors and emotional responding, due to the starting point differing substantially from PTEs. Even within an escape extinction treatment protocol, the clinician can wait until the child is ready to participate. The session would not terminate if the child decides they are not yet ready to participate; instead, the clinician could offer choices for when they are ready to participate (e.g., 2 min or 5 min) and how they would like to participate. However, because escape continues to be available in this example, it is possible that this form of escape extinction may not result in progress towards consumption.

The inclusion of a more traditional nonremoval procedure may be warranted where the spoon must be held to the child's mouth until tolerance and cooperation with the feeding instruction occurs. It is critical that data are collected on all behaviors during feeding treatment, including gagging to understand if treatments are effective and if refusal behaviors and emotional responding are decreasing. In Gover et al. (47), gagging occurred for 2 of the 3 children; however, it was unclear how this was addressed and whether the gagging and its function were assessed prior to treatment. It may also be useful to collect indices of happiness data to ensure that trauma-informed care practices are being utilized (81) and treatment changes are made if indices of happiness continue to decrease. Therefore, if we measure these types of behaviors during treatment, we can better understand the impact of the intervention on the child and whether treatments need to be altered to avoid PTEs.

Even if some form of escape extinction is required, it can be implemented with a preferred utensil, less aversive food type, or small bite size, for example, to increase the likelihood of contacting automatic and positive reinforcement (28, 29). As interfering behaviors decrease and the child contacts positive reinforcement with food and the eating situation, food engagement and consumption will increase over time.

## Conclusion and future direction

We propose a novel treatment framework integrating trauma-informed care practices with evidence-based behavioral treatment for pediatric feeding problems. It is important to note that the recommendations provided in this paper are not limited to children with identified trauma histories, but are broadly applicable across the pediatric feeding population. The framework is grounded in a least-restrictive hierarchy, in which more restrictive procedures are pursued only when necessary to achieve clinically meaningful outcomes, consistent with Austin et al. (10).

The research on trauma-informed care and behavioral feeding treatment is in its infancy, but understanding an individual's possible trauma history can help with the development of appropriate individualized assessments and treatments. However, the extensive empirical evidence demonstrating the efficacy of escape extinction should not be dismissed (26, 33), yet we also cannot disregard new findings. The use of escape extinction and trauma-informed care practices do not have to be dichotomous (11).

This practical framework allows clinicians to treat children using a continuum, where antecedents are evaluated first along with providing choices when appropriate while taking into account a child's unique history. Future studies need to determine whether systematically evaluating the starting point for treatment while taking into account current feeding repertoire and skill level within a trauma-informed framework leads to long-term treatment outcomes which focus on (1) tube removal, (2) weight management, (3) consuming a nutritionally balanced diet, (4) consuming age-appropriate portions, (5) independent eating, (6) positive relationship with food, (7) generalization to natural settings, (8) long-term maintenance, (9) improved parent-child relationship, and (10) reduced caregiver stress.

Gover et al. (47, 48) conducted extensive assessments prior to treatment that were somewhat aligned with trauma-informed care practices. However, in one of the studies, food selection was still determined arbitrarily based on caregiver chosen foods (47), rather than understanding why foods were deemed preferred or nonpreferred and using this information to guide treatment. In Gover et al. (48), the participant selected the foods to target in treatment, but it was unclear how the participant selected these foods without any previous exposure or knowledge about the food. Although the authors provided a framework to use trauma-informed care practices by incorporating choice, assent, and participant social validity, long-term success beyond 4 months was not provided, nor were generalization data presented from a typical meal in the natural setting or with foods not targeted in treatment (48). Post-treatment preference assessment data were also not collected; therefore, it is unclear whether preference actually developed for target foods and if the children were in fact eating a well-balanced diet at the end of treatment without a treatment plan, which would be the ultimate goal of any treatment program. The same can be true for escape extinction outcomes. Although escape extinction has demonstrated efficacy with respect to behavioral improvements, further research is needed to evaluate its effects on long-term health measures and social and emotional functioning (36, 37).

Some researchers in the area of trauma-informed care and feeding deem escape extinction restrictive and are proposing an alternative for treatment despite its demonstrated efficacy. However, it is likely that trauma-informed care practices can be implemented in conjunction with escape extinction if a socially significant change in behavior is not obtained without escape extinction. We would be doing an injustice to the patients we serve if we assume escape extinction is not appropriate or necessary, especially when we are working with a potentially life-threatening problem.

Prioritizing a child's autonomy is important; however, assent may not always be feasible. For example, children receiving tube feedings are not providing assent to be fed through the tube necessarily; rather the caregiver has to administer the feedings for the health and well-being of their child. A child may have trauma associated with being fed via tube, potentially due to tube feeding under physical restraint (13), vomiting after tube feedings, or an infection around the tube. Regardless, the child does not have the choice to be fed or not to be fed. The work by Gover et al. (48) represents an important step toward emphasizing trauma-informed care practices in feeding treatment and hopefully encourages feeding researchers, including those already implementing such practices, to explicitly report these steps in published research and to demonstrate long-term treatment outcomes.

If we are trying to achieve long-term meaningful change in the lives of children with feeding problems, some form of escape extinction may be necessary initially and full autonomy at the onset of treatment may not be possible until the child starts to contact positive reinforcement. Furthermore, the constraints imposed by health insurance coverage may necessitate the implementation of the most effective evidence-based intervention available to maximize treatment success while promoting the child's overall well-being and maintaining adherence to trauma-informed care principles.

Unfortunately, children do not always have a choice in their medical treatment, especially if it is a life-or-death situation and feeding may fall within this category. That being said, it is still important that a child's history be taken into account prior to treatment development and the least intrusive methods should be attempted first. However, if escape extinction is implemented with the least aversive stimulus condition, then it may appear to be less negative from the child's perspective (28, 29). If in fact it is less aversive, the motivation to escape may decrease and consumption may increase. The child may also be less likely to be retraumatized during the initial treatment because the stimulus conditions and reinforcement contingencies do not resemble PTEs. Future research needs to conduct long-term outcome studies evaluating the trauma-informed care model proposed in this paper for feeding treatment and collecting social validity measures from both caregivers and children.

## Data Availability

The original contributions presented in the study are included in the article/Supplementary Material, further inquiries can be directed to the corresponding author/s.
